# Induction of apoptosis by the retinoid inducible growth regulator RIG1 depends on the NC motif in HtTA cervical cancer cells

**DOI:** 10.1186/1471-2121-10-15

**Published:** 2009-02-26

**Authors:** Fu-Ming Tsai, Rong-Yaun Shyu, Su-Ching Lin, Chang-Chieh Wu, Shun-Yuan Jiang

**Affiliations:** 1 Department of Research, Buddhist Tzu Chi General Hospital Taipei Branch, Taipei county 231, Taiwan, Republic of China; 2 Department of Microbiology, Soochow University, Shih Lin, Taipei 111, Taiwan, Republic of China; 3 Department of Internal Medicine, Buddhist Tzu Chi General Hospital Taipei Branch, Taipei county 231, Taiwan, Republic of China; 4 Department of Surgery, Tri-Service General Hospital, Taipei 114, Taiwan, Republic of China

## Abstract

**Background:**

Retinoid-inducible gene 1 (RIG1), also known as tazarotene-induced gene 3 or retinoic-acid receptor responder 3, is a growth regulator, which induces apoptosis and differentiation. RIG1 is classified into the NC protein family. This study investigated functional domains and critical amino acids associated with RIG1-mediated cell death and apoptosis.

**Results:**

Using enhanced green fluorescence protein (EGFP)-tagged RIG1 variants, RIG1 proteins with deletion at the NC domain significantly decreased cell death induced by RIG1, and fusion variants containing only the NC domain significantly induced apoptosis of HtTA cervical cancer cells. The EGFP-RIG1-induced apoptosis was significantly decreased in cells expressing N^112^C^113 ^motif double- (NC→FG) or triple- (NCR→FGE) mutated RIG1 variants. Using dodecapeptides, nuclear localization and profound cell death was observed in HtTA cells expressing wild type RIG1_111–123 _or Leu^121^-mutated RIG1_111–123_:L→ C peptide, but peptides double- or triple-mutated at the NC motif alone, RIG1_111–123_:NC→FG or RIG1_111–123_:NCR→FGE, were cytoplasmically localized and did not induce apoptosis. The RIG1_111–123 _also induced apoptosis of A2058 melanoma cells but not normal human fibroblasts.

**Conclusion:**

The NC domain, especially the NC motif, plays the major role in RIG1-mediated pro-apoptotic activity. The RIG1_111–123 _dodecapeptide exhibited strong pro-apoptotic activity and has potential as an anticancer drug.

## Background

*Retinoid-inducible gene 1 (RIG1*) [1], which encodes a protein of 164 amino acids also known as *tazarotene-induced gene 3 *(*TIG3*) [2] or *retinoic-acid receptor responder 3 *(*RARRES3*) [3], belongs to the HREV107 gene family that contains five members in humans [4-6]. Proteins of the HREV107 family share four conserved domains, a proline-rich motif located at the N-terminus, followed by a conserved H-box, the NC domain and a C-terminal transmembrane domain [6-8]. The HREV107 family proteins are growth regulators, and have recently been classified into the NC protein family along with the lecithin:retinol acyltransferase (LRAT), protein 2A from Avian encephalomyelitis virus and Echovirus 22 and a hypothetical protein At5g16360 from Arabidopsis thaliana [6,8]. HREV107 family proteins are typically localized within the endomembranes, such as the endoplasmic reticulum (ER) and Golgi apparatus [9-13]. However, nuclear localization has been reported for the HREV107 proteins [11,14].

RIG1 exhibits growth suppressive and proapoptotic activities in normal keratinocytes [13] and cancer cells of various origins [10,12,15,16]. The proapoptotic activities of RIG1 are mediated through caspase-dependent [12] or -independent pathways [13], and are initiated only by the Golgi-, but not the ER-targeted RIG1 [12]. In addition, RIG1 stimulates cellular differentiation of keratinocytes, which is mediated by the activation of type I tissue transglutaminase [13,17,18]. Also, RIG1 inhibits the signaling pathways of Ras and phosphoinositide-3 kinase (PI3K)/serine/threonine-specific protein kinase (AKT) [12,19,20].

The structure/function relationship of RIG1 has been investigated. Presence of the C-terminal transmembrane domain, ranging from 134 to164 amino acids, that targets the protein to endomembranes is essential for RIG1-mediated activities [10,12,13,17-19]. The RIG1 segment spanning amino acids 124–164 interacts with and serves as the substrate of tissue transglutaminase I [18]. Also, the N-terminal 124 amino acid region is required for RIG1-dependent keratinocyte differentiation, and removal of the region converts RIG1 into a proapoptotic protein in human keratinocytes. Within the NC domain, the Asp^112^-Cys^113 ^(NC) motif of RIG1 is the most conserved feature throughout evolution from virus to *Homo sapiens *[7], and the Cys^161 ^of LRAT, corresponding to the Cys^113 ^of the RIG1, is proposed to participate in a catalytic triad that is directly involved in the LRAT-catalyzed esterification [21]. Recently, dodecapeptides (DPs) H-TIG-3_111–123 _and H-Ha-Rev107-1_111–123_, based on 12 conserved amino acids surrounding the NC motif within the NC domain of RIG1 and HREV107, have been shown to induce apoptosis of melanoma cells, and the Leu^120 ^has been shown to be indispensable for this activity [22]. The H-TIG-3_111–123_, which is referred to as RIG1_111–123 _hereafter, is targeted to the nucleus where it binds and activates promoters of transcription factors involved in the G1 → S transition. The nuclear localization and DNA binding activities of RIG1_111–123 _sharply contrasts with the character of wild type RIG1 that is predominantly localized in the cytoplasm and binds to RAS [12,19] and transglutaminase [13,17,18]. The function of the proline-rich region and H-box of RIG1 has not been investigated, although the proline-rich region of HREV107 has recently been shown to bind PR65α, a subunit of the protein phosphatase 2A, and to inhibit the enzyme's activity [23].

This study investigated functional regions and critical amino acids that are essential for the proapoptotic activities of RIG1 in HtTA cervical cancer cells. Both full-length RIG1 and various truncated variants were constructed with an enhanced green fluorescence protein (EGFP) tag to the N-terminus of the protein. Because RIG1_111–123 _also functions as an anti-proliferative peptide [22], and to further evaluate critical amino acids responsible for RIG1_111–123_- and RIG1-mediated activities, wild type and mutated (Asp^112^, Cys^113^, Leu^120 ^and Arg^121^) DPs as well as the full length RIG1 containing various mutations tagged to the C-terminal of EGFP were constructed. The NC motif was selected due to its evolutionary conservation from virus to mammalian [7]. Leu^120 ^is shown to play a critical role in the RIG1_111–123_-mediated activities in E:WM-115 melanoma cells, and Arg^121 ^may play a role involving DNA contacts [22]. Therefore, Leu^120 ^and Arg^121 ^were selected for the analysis. The results demonstrate that the NC motif of RIG1 is important for RIG1-mediated cell death. In addition, the NC motif, but not Leu^120 ^is essential for RIG1_111–123_-mediated induction of cell death of HtTA cells.

## Results

### The NC domain is important for RIG1 in the induction of cell death and apoptosis

**Figure 1:**
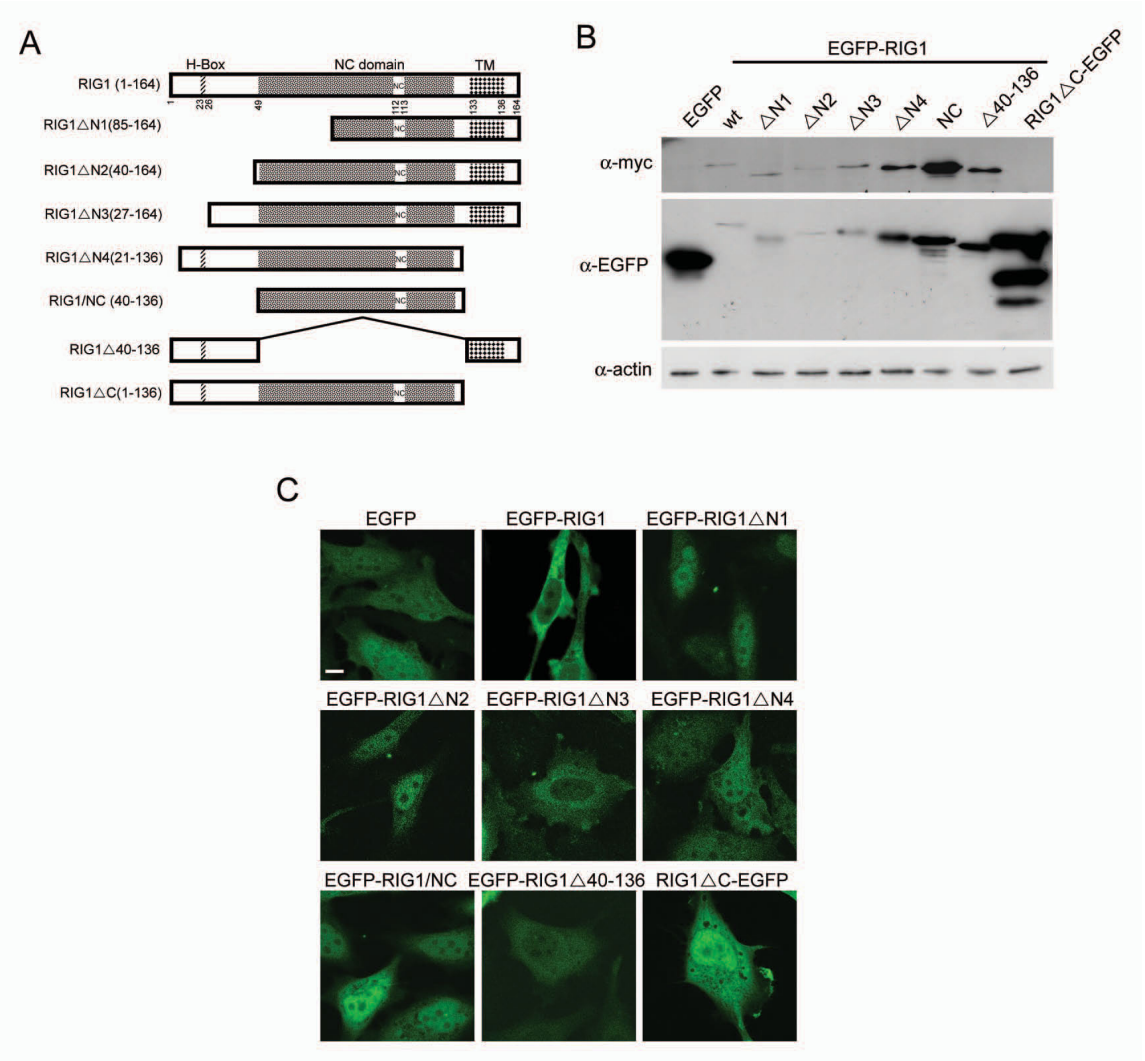
**Analysis of the expression of various truncated EGFP-RIG1 fusion proteins**. (**A) **Schematic diagram of RIG1 expression vectors that express wild type or various truncated RIG1 proteins. (**B) **Western blot analysis of the truncated EGFP-tagged RIG1 fusion proteins. HtTA cells plated in 6-cm dishes were transiently transfected with 1.5 μg of indicated expression vectors for 24 h. Fifty μg of cellular proteins were resolved by 15% SDS-PAGE. Expression of RIG1 variants was detected using anti-myc or anti-EGFP antibodies. (**C) **Subcellular localization of EGFP-tagged RIG1 variants. Cells were transiently transfected with indicated expression vectors for 18 h. Cells were fixed and analyzed with a laser scanning confocal microscope. Scale bar: 5 μm; wt: wild type.

To identify functional domains of RIG1 which are related to induction of cell death or apoptosis, we first constructed expression vectors that synthesized recombinant proteins containing EGFP tagged to the N-terminus of the wild type RIG1 or various truncated RIG1 variants, with the exception of RIG1ΔC in which EGFP was tagged to the C-terminus of the RIG1 (Fig 1A). The RIG1ΔN1, RIG1ΔN2, RIG1ΔN3 and RIG1ΔN40–136 variant proteins all contained the membrane spanning domain but differed in the H-box and NC domains. The C-terminal deleted RIG1ΔN4, RIG1/NC and RIG1ΔC proteins vary in the N-terminal 40 amino acids including the H-box. Expression of EGFP-tagged RIG1 variants at the expected molecular weight was demonstrated in transfected cytosols prepared from HtTA cervical cancer cells (Fig 1B). The EGFP-tagged wild type and N-terminal truncated (RIG1ΔN1, RIG1ΔN2, and RIG1ΔN3) RIG1 recombinant proteins were expressed at much lower levels compared to that of variants without C-terminal hydrophobic domain. Similar results were also observed in previous studies [12,18,19]. With the exception of RIG1ΔN1 and RIG1ΔN2, which had mainly nuclear localization with weak and diffuse cytoplasmic distribution, all the EGFP-tagged recombinant proteins that contained the C-terminal hydrophobic domain and the N-terminal region between 27 and 39 amino acids (RIG1, RIG1ΔN3 and RIG1Δ40–136) were primarily distributed in the perinuclear region (Fig 1C), and were partially co-localized to the Golgi apparatus [see Additional file 1]. The C-terminal truncated RIG1 with EGFP tagged to either the C- (RIG1ΔC-EGFP) or N-terminus [10] were diffusely expressed throughout the nucleus and cytoplasm. Variants (EGFP-RIG1ΔN4 and EGFP-RIG1/NC) that had truncation at both the N- and C-termini exhibited similar expression patterns as the RIG1ΔC-EGFP with the exception of perinuclear localization of the fusion proteins also detected in less than 5% of cells.

**Figure 2:**
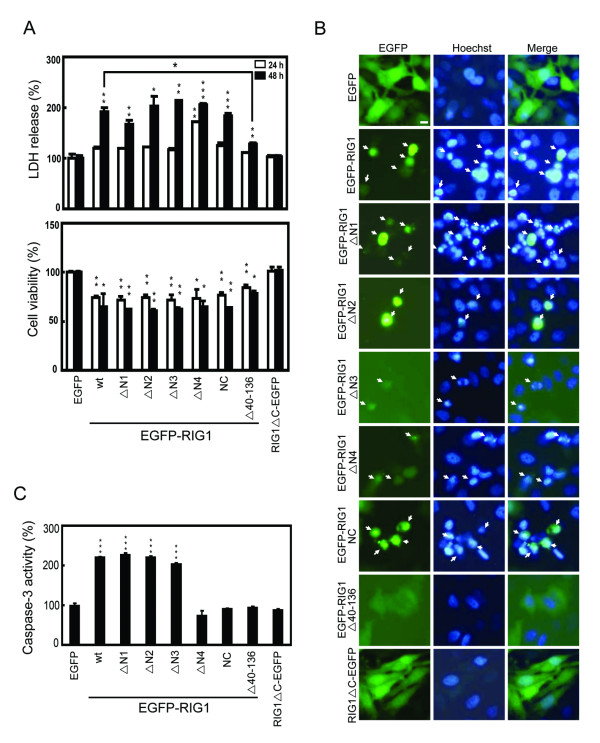
**Truncated EGFP-RIG1 fusion proteins induced cell death and apoptosis**. **(A) **HtTA cells were transfected with 0.3 μg of indicated recombinant RIG1 expression vectors for 24 or 48 h. Cell death was detected by measuring LDH release and cell viability was measured using the MTT method. Representative results of three independent experiments are shown and are expressed as means and standard errors of the means after normalization to the control group. (**B) **Cells were transfected with indicated expression vectors for 48 h. Cells were stained with Hoechst 33258 dye, and chromatin condensation was evaluated with a fluorescent microscope. Arrows indicate cells expressing the EGFP or RIG1 fusion protein that were positive for chromatin condensation. Scale bar: 1 μm. (**C) **Cells were transfected with indicated expression vectors for 24 h. The activation of caspase-3 was assayed using the colorimetric substrate Ac-DEVD-pNA. wt: wild type; Student *t *test: *, P < 0.05; **, P < 0.01; ***, P < 0.001.

Cell death and viability, determined by measuring the release of lactate dehydrogenase (LDH) and by the MTT assay respectively, were then analyzed in HtTA cells transiently transfected with various deleted EGFP-RIG1 expression vectors. With the exception of RIG1ΔC-EGFP that had no effect on cell death and cell viability, wild type and the other EGFP-RIG1 deletion variants significantly increased cell death by 29%–115% and reduced cell viability by 22–39% after transient expression for 48 h (Fig 2A), and the EGFP-RIG1Δ40–136, which lacks the NC domain, had the weakest effect on both cell death and cell viability among the RIG1 fusion proteins tested. In contrast, the EGFP-tagged RIG1 variant that contained only the NC domain (EGFP-RIG1/NC) significantly increased cell death by 82% and reduced cell viability by 36%. Similar effects of wild type and truncated RIG1 variants on cell growth were also observed in HtTA cervical cancer cells expressing the monomer red fluorescence protein (MRFP)-tagged recombinant RIG1 variants (data not shown), indicating the effects on cell growth was mediated by RIG1 fragment within the recombinant protein. The effects of EGFP-tagged recombinant RIG1 variants on cell death were further explored by the analysis of chromatin condensation in HtTA cells 48 h after transfection. Chromatin condensation was readily detected in most cells expressing the EGFP-tagged wild type or truncated RIG1 with the exception of RIG1ΔC-EGFP and EGFP-RIG1Δ40–136 (Fig 2B). These results suggest that the NC domain plays the major role in the growth inhibition and proapoptotic activities of RIG1.

Whether induction of cellular apoptotosis by RIG1 variants in HtTA cells was related to caspase 3 activation was then analyzed in cytosol extracts prepared 24 h after transfection. Caspase 3 activities in cytosol extracts prepared from EGFP-tagged recombinant RIG1 variants that contained the C-terminal hydrophobic domain (RIG1, RIG1ΔN1, RIG1ΔN2 and RIG1ΔN3) were significantly enhanced ranging from 205 to 227% of control levels (Fig 2C). However, RIGIΔC-EGFP, EGFP-RIG1Δ40–136, EGFP-RIG1ΔN4 and EGFP-RIG1/NC did not activate caspase 3 in HtTA cells.

### The NC motif plays a critical role in DP-induced cell death and apoptosis

**Figure 3:**
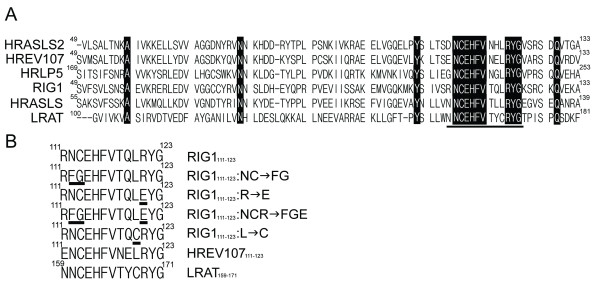
**Designation of mutated DP of HREV107 family protein**. **(A) **Sequence alignment of the NC domains of human HREV107 family proteins and LRAT. Identical amino acids are indicated by black boxes and the DP is underlined. (**B) **Comparison of amino acid sequences of DP and DP variations (changed amino acids underlined) used in this study. Sequences of DPs derived from HREV107 and LRAT are also indicated.

**Figure 4:**
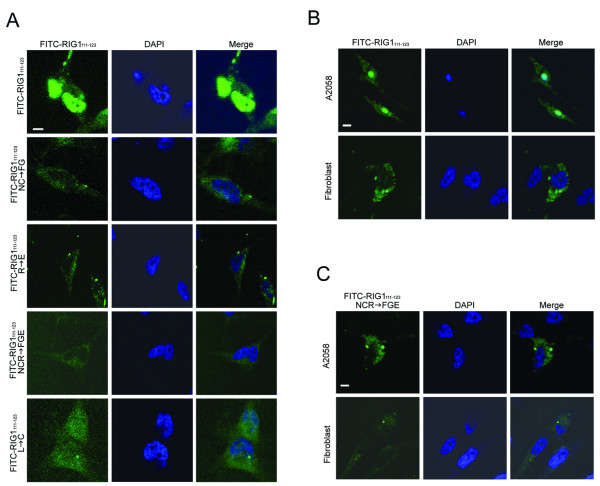
**Subcellular localization of FITC-DPs**. HtTA **(A)**, A2058 cells and normal human fibroblasts (B and C) plated in 6-well plates overnight were electroporated with 5 μM of the indicated FITC-labeled DPs and then incubated for 48 h. Cells were fixed, stained with DAPI, and analyzed with a laser scanning confocal microscope. Scale bar: 5 μm.

A multiple alignment of amino acid sequences of the NC domain among the HREV107 family proteins and LRAT is shown in Fig 3A. Thirteen amino acids within the NC domain are identical among all 6 members, and the DP region exhibits the highest degree of homology. Fluorescein (FITC)-labeled wild type and mutated RIG1_111–123 _peptides (Fig 3B) were introduced into HtTA cells to analyze activities and critical amino acids of DP affecting cell death. The wild type FITC-RIG1_111–123 _was predominantly distributed within the nucleus of HtTA cells 48 h after electroporation (Fig 4A). The Leu^120^-mutated FITC-RIG1_111–123_:L → C was distributed homogenously between cytoplasm and nucleus. In contrast, DPs containing mutation at one (R), two (NC) or three (NCR) amino acids were all cytoplasmically localized. Similar nuclear and cytoplasm distribution of wild type and NCR-mutated RIG1_111–123 _respectively was observed in A2058 melanoma cells. However, both the wild type and NCR-mutated RIG1-DPs were distributed only within the cytoplasm of normal human fibroblasts (Fig 4B, C). In addition to nuclear targeting, RIG1_111–123 _also induced nuclear shrinkage, a characteristic of cellular apoptosis, of A2058 cells.

**Figure 5:**
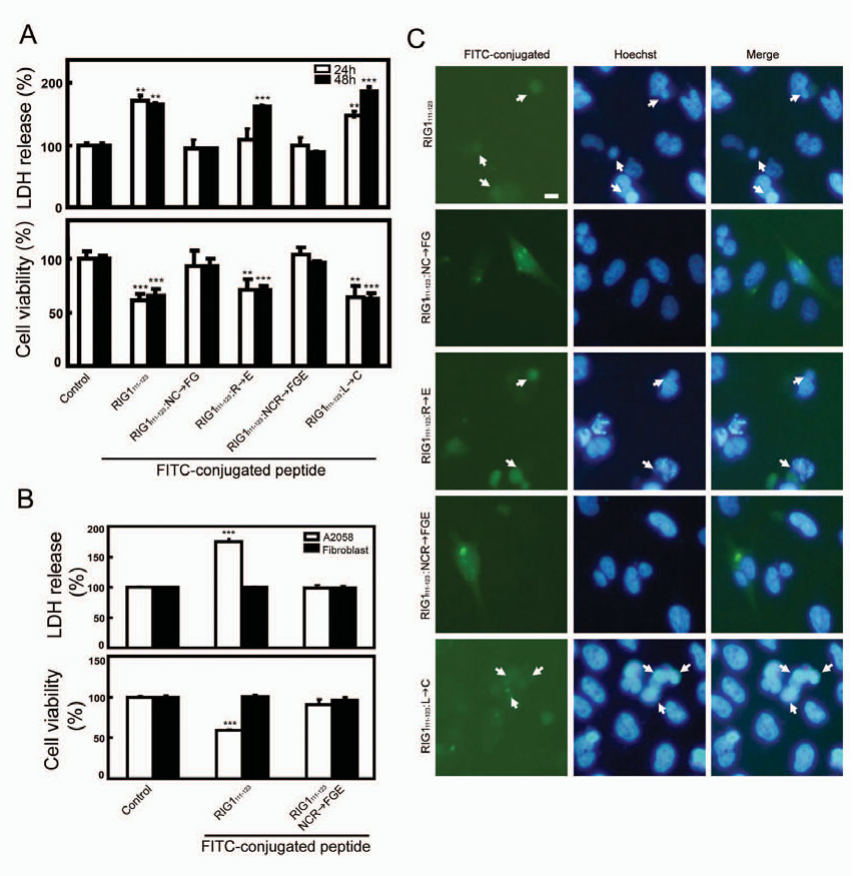
**FITC-DPs induced cell death and apoptosis**. HtTA (A and C), A2058 (B) cells and normal human fibroblasts (B) were electroporated with 5 μM of the indicated FITC-labeled DPs or with medium only (control). Cells were washed and incubated for 24 and 48 (A) or for 48 (B and C) h. Cell death was detected by measuring LDH release and cell viability was measured with the MTT method (A and B). Representative results of three independent experiments are shown and are expressed as means and standard errors of the means after normalization to the control group. Student *t *test: **, P < 0.01; ***, P < 0.001. (**C) **Cells were stained with the Hoechst 33258 dye, and chromatin condensation was evaluated with a fluorescent microscope. Arrows indicate that cells expressing the FITC-conjugated peptide and were positive for chromatin condensation. Scale bar: 5 μm.

Analysis of FITC-DPs effect on cell death showed that wild type FITC-RIG1_111–123 _significantly increased cell death by 67% and decreased cell viability by 34% 48 h after peptide introduction in HtTA cells, and the effects were also significantly evident 24 h after peptide introduction (Fig 5A). Similarly, the Leu^120 ^or Arg^121 ^mutated FITC-DPs both significantly increased cell death by 64–89% and decreased cell viability by 29–37% 48 h after peptide introduction. FITC-DPs that contained two (NC) or three (NCR) amino acid mutations had no effect on cell death and cell viability. Unrelated peptide (FITC-GYFHEGFHGYFGY) [22] was also tested for the activity on cell death and cell viability 24 and/or 48 h after peptide introduction, and no effect was observed (data not shown). Similar effects on cell death and viability mediated by the wild type but not NCR-mutated FITC-RIG1_111–123 _were observed in A2058 melanoma cells but not in normal human fibroblasts (Fig 5B). Activities of FITC-DPs on chromatin condensation were also analyzed in HtTA cells. Chromatin condensation was observed only in cells electroporated with wild type, Leu^120^-mutated or Arg^121^-mutated FITC-DPs, but not in cells with NC- or NCR-mutated FITC-DPs (Fig 5C). Similarly, fusion protein containing RIG1_111–123 _tagged to the C-terminus of EGFP (EGFP-RIG1_111–123_) [see Additional file 2] induced cellular chromatin condensation, and significantly induced cell death of HtTA cells as determined by MTT and LDH assay [see Additional file 3], and the RIG1_111–123_-mediated cell death and apoptosis is reversed by mutation at the NC motif, but not at Leu^120 ^or Arg^121 ^within the EGFP-tagged DP. Finally, cytosol extracts prepared from HtTA cells electroporated with FITC-conjugated DPs for 24 h were analyzed for caspase 3 activation, and no caspase 3 activation was detected in all cytosol extracts (data not shown).

### Mutation at the NC motif reduces RIG1-mediated cell death

**Figure 6:**
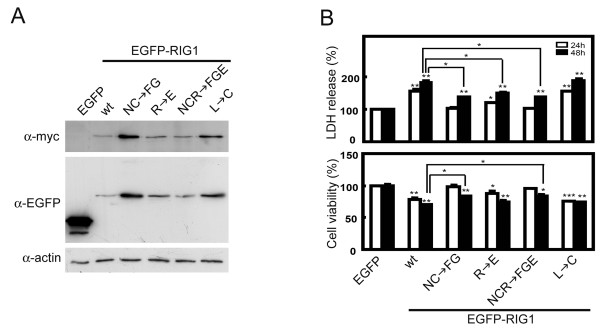
**Analysis of the expression and effects on cell growth of various mutated EGFP-RIG1 fusion proteins**. **(A) **Western blot analysis of the mutated EGFP-RIG1 proteins. HtTA cells plated in 6-cm dishes were transiently transfected with 1.5 μg of indicated expression vector for 24 h. (**B) **Mutated EGFP-RIG1 fusion proteins induced cell death. HtTA cells were transfected with 0.3 μg of the control EGFP or indicated EGFP-tagged wild type or mutated RIG1 expression vectors for 24 or 48 h. Cell death was detected by measuring LDH release, and cell viability was measured with the MTT method. Representative results of three independent experiments are shown and are expressed as means and standard errors of the means after normalization to the control group. wt, wild type; Student *t *test: *, P < 0.05; **, P < 0.01; ***, P < 0.001.

To further characterize the significance of amino acids within the DP in RIG1-mediated cell death and cell viability in HtTA cells, we constructed constitutive EGFP-RIG1 expression vectors that synthesized wild type RIG1 or RIG1 with single (R → E or L → C), double (NC → FG) or triple (NCR → FGE) amino acid substitution. Proper expression of mutated EGFP-RIG1 fusion proteins in HtTA cells was confirmed by Western blotting (Fig 6A). Expression of wild type EGFP-RIG1 for 24 or 48 h resulted in significant increase of cell death by 57 or 83%, respectively, and reduction of cell viability by 22 or 29%, respectively (Fig 6B). Expression of Leu^120 ^substituted EGFP-RIG1 for 24 or 48 h resulted in significant increase of cell death by 56 or 89%, respectively, and reduction of cell viability by 24 or 26%, respectively. Mutation at Leu^120 ^did not reduce EGFP-RIG1-mediated cell death or cell viability. Significant effects of the Arg^121 ^substituted EGFP-RIG1 on cell death and cell viability were observed both at 24 and 48 h after transfection, although the effect was weaker than that of EGFP-RIG1. Increase of cell death and decrease in cell viability in EGFP-RIG1 variant transfectants containing double (NC → FG) or triple (NCR → FGE) mutations were not evident until 48 h after transfection with maximal 37% increase of cell death and 17% reduction of cell viability. The effects of double or triple amino acid substitutes were significantly less than the effects induced by EGFP-RIG1, indicating that the NC motif of RIG1 is important for the effects of RIG1 on cell death. Similar to the results shown in the Fig 5C, chromatin condensations as observed in cells expressing the EGFP-RIG1 were profoundly decreased in cells expressing double (NC → FG) or triple (NCR → FGE) mutated EGFP-RIG1 variants. Leu^120 ^and Arg^121 ^substitution had no effect on or had weakly reduced EGFP-RIG1-induced chromatin condensation of HtTA cells (data not shown).

## Discussion

Through expression of EGFP-tagged RIG1 variants, we have identified that the NC domain, especially the NC motif, plays the most profound role in the RIG1-mediated growth inhibition and cellular apoptosis of HtTA cells. The conclusion is supported first by significantly decreased the effect of RIG1 on cell death of the fusion protein (RIG1Δ40–136) with deletion at the NC domain, and fusion proteins that contain only the NC domain (RIG1/NC) exhibit profound proapoptotic activity. In addition, we demonstrated that the RIG1_111–123 _within the NC domain increases cell death of HtTA cells, and that mutation of the NC motif results in complete abolishment of this activity. The importance of the NC motif is further demonstrated by the significant decrease of cell death in cells expressing the recombinant RIG1 variants that have two (NC) or three (NCR) amino acid mutations. Significance of the NC motif in RIG1 function is supported by the evolutionary conservation of the motif [6,7]. Although a decrease of RIG1 expression was frequently observed in several types of cancer tissues [24-27], no mutation at the NC motif of RIG1 has been reported.

Analysis of the structure/function relationship of the NC motif and adjacent amino acids has been very limited. Mondal and coworkers first demonstrated that both cysteine residues within the DP of LRAT (LRAT_159–171_) (Fig 3B) are important for its enzymatic activity, and that the cysteine in the NC motif is the critical active-site involved in catalysis [28]. Consistent with previous observations that demonstrate proapoptotic activities of DPs derived from HREV107 and RIG1 in melanoma cells (Fig 4B) [22], we show that RIG1_111–123 _induces cell death when introduced into HtTA cervical cancer cells as either a FITC-conjugated peptide or an EGFP fusion protein. In contrast to the significant role of Leu^120 ^in RIG1_111–123_-mediated growth inhibition of E:WM-115 melanoma cells [22], this study demonstrated that the NC motif is indispensable, the Arg^121 ^residue plays a minor role and the Leu^120 ^has no effect on the anti-proliferative activities of RIG1_111–123 _in HtTA cells. This conclusion is supported first by FG for NC substitution which resulted in complete or significantly loss of anti-proliferative activities of FITC-RIG1_111–123 _or EGFP-RIG1_111–123 _respectively, second by profound loss of anti-proliferative activities in NC or NCR mutated RIG1 fusion proteins, third by reduced proapoptotic activities of the Arg^121^mutated RIG1_111–123 _and RIG1, and fourth by the lack of effect of Leu^120 ^mutation on proapoptotic activities induced by RIG1_111–123 _or RIG1. In addition to HtTA cervical cancer cells, results from this and previous [22] studies also show growth suppressive activities of RIG1_111–123 _on established or primary culture of melanoma cells but not normal human fibroblasts and proliferating melanocytes. Therefore, RIG1_111–123 _is selectively toxic to tumor cells, and the significance of Leu^120 ^in the RIG1_111–123_-mediated growth suppression shown previously in melanoma cells is tumor cell type specific.

RIG1_111–123 _is predicted to have an alpha helical structure [6,22], and selectively binds to cis-binding elements and activates transcription factors involved in the G1/G0 → S transition in melanoma cells [22]. In this study, we demonstrated that RIG1_111–123 _is targeted to nuclei of sensitive HtTA and A2058 cells, and mutations that render the loss of growth inhibitory activities result in cytoplasmic distribution of the DPs. Also, RIG1_111–123 _that has no effect on normal fibroblasts is distributed only within the cytoplasm. The tight association between nuclear localization and growth suppressive activities of RIG1_111–123 _supports the notion that RIG1_111–123_-mediated cell death in sensitive tumor cells is likely to be mediated through sequence-specific DNA binding that leads to alteration in gene expression involved in the cell cycle and apoptosis. Absence of nuclear localization of RIG1_111–123 _in resistant melanoma cells [22] and normal human fibroblasts may be related to failure in nuclear trafficking or to retention of the peptide. The α-helical structure of RIG1_111–123 _within the EGFP-RIG1_111–123 _fusion protein is mostly maintained predicted using SSpro and PSIPREDView programs. This supports the growth suppressive activity of EGFP-RIG1_111–123_. In contrast to complete loss of activities in NC- or NCR-mutated DPs, growth suppressive activities of the NC- or NCR-mutated EGFP-DP fusion proteins were significantly, but not completely, reduced. The increase in nuclear localization of the double and triple mutated EGFP-DPs, as compare to that of FITC-conjugated DPs, may be related to partial growth suppressive activities of the mutated EGFP-DPs. Currently, critical amino acids involving the binding of RIG1_111–123 _to cis transcription elements have not been addressed, although RIG1_111–123 _has been shown to bind cis binding elements for receptors of retinoic acids [22]. The fact that nuclear distribution of RIG1_111–123 _was completely disrupted by mutations at the NC motif or Arg^121^, and was only partially altered by Leu^120 ^mutation suggests that the NC motif and Arg^121 ^play a profound role and Leu^120 ^a minor role in the binding of RIG1_111–123 _to cis-elements. In addition to the four amino acids investigated in this study, six amino acids (EHFV–YG) within RIG1_111–123 _are conserved across the HREV107 protein family and LRAT (Fig 3A). Further analysis using site-directed DP mutants will be useful in dissecting the role of the NC motif, Arg^121^, Leu^120 ^and other six conserved amino acids in the DNA binding properties as well as regulation of promoter and growth suppressive activities of RIG1_111–123_.

RIG1ΔN4 and RIG1ΔC are different only by 20 amino acids at the N-terminus, which consist of the proline-rich region. RIG1ΔN4 that is devoid of the proline-rich region inhibits growth and induces apoptosis of HtTA cells. However, RIG1ΔC fails to have effect on cell growth, apoptosis and activation of Ras and tissue transglutaminase I when the truncated protein is expressed as fusion proteins that have myc or EGFP tagged to the N- or C-terminus [10,12,13,18,19]. These results suggest that the N-terminal proline-rich region of RIG1 may bind effectors that prevent RIG1ΔC from exerting effects on the regulation of cell growth and apoptosis. Truncation of the N-terminal region, as demonstrated in RIG1ΔN4, restores these activities. In fact, the proline-rich region of HREV107 has recently been shown to bind a subunit of the protein phosphatase 2A, PR65α. The binding leads to inhibiting the enzyme's activity and is important for proapoptotic activities of HREV107 on ovarian cancer cells [23]. Identification of a binding protein for the proline-rich region of RIG1 will be important to delineate the functional domain at the extreme N-terminus of RIG1.

Most results from previous studies demonstrate perinuclear and endomembrane localization of transfected wild type RIG1, HREV107 and HRASLS, which induce cellular apoptosis and/or differentiation of normal and cancer cells [9,12-14,18]. However, nuclear localization of HREV107 and RIG1 was also observed in fibroblasts and cancer cells, and cytoplasmic but not nuclear targeted HREV107 is related to growth stimulation and poor patient survival of non-small cell lung cancer [11,14,24]. Therefore, proteins of this gene family may localize to multiple intracellular compartments where they have distinct activities. Previous studies in HtTA cells and keratinocytes have demonstrated that RIG1-induced apoptosis or differentiation-related apoptosis is mediated through Golgi apparatus or mitochondria via caspase 3-dependent and/or -independent mechanisms [12,13]. Similarly, caspase 3-mediated apoptosis is reported in keratinocytes expressing RIG1 with 124 amino acids truncated at the N-terminus [18]. Results of this study in HtTA cells also demonstrated activation of caspase 3 during apoptosis induced by variants (RIG1ΔN1, RIG1ΔN2 and RIG1ΔN3) that contain the C-terminal hydrophobic domain but not RIG1ΔN4 and RIG1/NC that are deleted at both N- and C-termini. Although caspase 3 activation was observed in RIG1ΔN1, RIG1ΔN2 and RIG1ΔN3, RIG1ΔN1 and RIG1ΔN2 are mainly distributed in the nucleus whereas RIG1ΔN3 is in the perinuclear region. Therefore, presence of C-terminal hydrophobic domain rather than subcellular localization appears to play an important role in the caspase 3 activation during apoptosis induced by N-terminal truncated RIG1 variants. The nuclear targeted RIG1_111–123 _that exhibits DNA binding and proapoptotic activities was first demonstrated by Simmons *et al*. [22]. This study further demonstrated that proapoptotic activities of truncated RIG1 variants, RIG1ΔN4 and RIG1/NC, and the nuclear targeted RIG1_111–123 _are mediated through the caspase 3-independent mechanism in cervical cancer cells. RIG1ΔN4 and RIG1/NC were found in both nucleus and cytoplasm. Whether the mechanism of apoptosis induced by RIG1ΔN4 and RIG1/NC is similar to that of RIG1_111–123 _that is mediated through regulation of gene expression, as proposed by Simmons et al [22], needs further investigation.

Wild type RIG1 and N-terminal truncated variants such as RIG1ΔN1, RIG1ΔN2 and RIG1ΔN3 were expressed at much lower levels than RIG1ΔN4 and RIG1/NC that were truncated at both N- and C-termini. Short protein half life [19] or an increase in distribution to the particulate fraction due to N-terminal truncation [18] may be responsible for the low protein levels observed in N-terminal truncated RIG1 variants. Although expressed at low levels, the extent of growth suppression on HtTA cells induced by N-terminal truncated variants was relatively similar to the RIG1ΔN4 and RIG1/NC. Similar potent proapoptotic activities, despite being expressed at extremely low levels, of RIG1 variants with N-terminal truncation for 112 to 124 amino acids were also reported in keratinocytes [18].

RIG1 is a growth regulator that suppresses cell growth through induction of cellular differentiation and/or apoptosis. The differentiation-inducing activity of RIG1 on keratinocytes is mediated through the activation of tissue transglutaminase I [13,17]. A structure/function analysis has demonstrated that the N-terminal region of RIG1 is required for pro-differentiation activities, regardless, the C-terminal hydrophobic region by itself is enough for the activation of tissue transglutaminase I [18]. Recently, results from our [12,19] and others [20] studies in HtTA cervical and ovarian cancer cells have demonstrated that RIG1 inhibits expression and activation of signaling molecules such as HER2, RAS, PI3K/AKT and mTOR that are involved in the regulation of cell growth, apoptosis and tumor invasion. Further analysis using various truncated and mutated RIG1 variants will be useful to dissect mechanisms as well as functional domains and critical motifs of RIG1 in the regulation of signal pathways involving growth factor receptors.

## Conclusion

In summary, RIG1 variants exhibit growth inhibitory activity both when localized to the nucleus and when localized to the cytoplasm. The fact that caspase 3 activation was detected in N-terminal truncated variants, such as RIG1ΔN1, RIG1ΔN2 and RIGΔN3, but not in C-terminal truncated variants supports the existence of multiple mechanisms during RIG1-mediated growth inhibition. The NC domain, especially the NC motif, plays an important role in RIG1-mediated apoptosis in HtTA cervical cancer cells. RIG1_111–123 _alone exhibits profound proapoptotic activities and appears to be useful for development as an anticancer drug.

## Methods

### Expression vectors

The vectors pRIG1ΔC-EGFP, pRIG1-myc and pRIG1ΔC-myc have been described previously [19]. Expression vectors (pRIG1ΔN1-myc, pRIG1ΔN2-myc, pRIG1ΔN3-myc and pRIG1ΔN4-myc, pRIG1Δ40–136) with deleted RIG1 variants containing myc and his epitopes were generated by site-directed mutagenesis using a single primer as described previously [29] (Fig. 1). Briefly, RIG1 deleted plasmids were constructed by amplification of the pRIG1-myc plasmid using primers for RIG1ΔN1 (5'-CAGAATTCCTTGGCTTCGAGATGCAACCACGGCCCGTGG-3'), RIG1ΔN2 (5'-CAGAATTCCTTGGCTTCGAGATGAGTGAGTACCCCGGGGCTG-3'), RIG1ΔN3 (5'-CAGAATTCCTTGGCTTCGAGATGTATATAGGAGATGGCTACGTGATCCATC-3') and RIG1ΔN40–136 (5'-GTGATCCATCTGGCTCCTCCAGTCGGTGTGGCCACGGCGCTTG-3'). The pRIG1ΔN4-myc plasmid was constructed by amplification of pRIG1ΔC-myc using the primer (5'-AGAATTCCTTGGCTTCGAGATGTATGAGCAATGGGCCCTGTATATA-3'). To generate expression vectors for EGFP-tagged (pEGFP-RIG1ΔN1, pEGFP-RIG1ΔN2, pEGFP-RIG1ΔN3, pEGFP-RIG1ΔN4, and pEGFP- RIG1Δ40–136) RIG1 variants, truncated RIG1 cDNA fragments were amplified from the respective myc- and his-tagged plasmids described above using 5' (5'-TCTCGAGTTCTTGGCTTCGAGATG-3') and 3' (5'-CGGAATTCTCAATGATGATGATGATG-3') primers, and then subcloned in-frame into *Xho*I-*Eco*RI sites of the pEGFP-C1 vector (Clontech Laboratories, Inc, Palo Alto, CA), respectively. The NC domain of the RIG1 cDNA fragment (RIG1/NC) was amplified from pRIG1ΔN2-myc using 5' (5'-AGAATTCCTTGGCTTCGAGATGAGTG-3') and 3' (5'-GTGGATCCTTCAACCTTGGCCTTTTC-3') primers and then subcloned in-frame into the pEGFP-C1 to generate pEGFP-RIG1/NC. To generate EGFP-tagged and RIG1 mutated expression vectors (pEGFP-RIG1:NC → FG, pEGFP-RIG1:R → E, pEGFP-RIG1:L → C), mutated RIG1 plasmids were generated via site-directed mutagenesis by amplification of the pEGFP-RIG1-myc using primers containing mutations at amino acids Asp^112 ^and Cys^113 ^(5'-CAGTATTGTGAGCAGGTTTGGCGAGCACTTTGTCACCCAGCTG-3'), Arg^121 ^(5'-CTTTGTCACCCAGCTGGAGTATGGCAAGTCCCGCTGTAAACAG-3'), and Leu^120 ^(5'-GCACTTTGTCACCCAGTGTAGATATGGCAAGTCCCGCTGTAAAC-3'). The pEGFP-RIG1:NCR → FGE was generated from pEGFP-RIG1:NC → FG using primer (5'-CTTTGTCACCCAGCTGGAGTATGGCAAGTCCCGCTGTAAACAG-3'). The amplified plasmids containing the indicated mutations were then digested with *Dpn*I and transformed into the *E. coli *HB101 strain. The cDNA sequences of RIG1 and the expression of fusion proteins were confirmed by DNA sequencing and Western blotting, respectively. Construction of expression vectors that synthesized MRFP tagged RIG1 variants and EGFP tagged DP recombinant proteins was described in supplementary material [see Additional file 4].

### Cell culture and transfection

HtTA cervical cancer cells (obtained from Dr. T.-C. Chang, Department of Biochemistry, National Defense Medical Center, Taiwan), A2058 melanoma cells and normal human fibroblasts (Food Industry Research and Development Institute, Taiwan) were maintained in RPMI-1640 medium supplemented with 25 mM HEPES, 26 mM NaHCO_3_, 2 mM L-glutamine, penicillin (100 units/ml), streptomycin (100 μg/ml), and 10% fetal bovine serum (FBS) at 37°C in 5% CO_2_. Cells plated in culture dishes were transfected with the expression vectors using liposome mediated-transfection. Briefly, plasmids and lipofectamine (Gibco BRL, Gaithersburg, MD) were diluted in Opti-MEM medium and then mixed with plasmids at room temperature for 15 min. The DNA-lipofectamine complexes were then added to cells for 2.5 h at 37°C. Cells were refreshed with complete medium for 24 h at 37°C for further analysis.

### Electroporation

Cells were plated on coverslips in six-well plates at 1 × 10^5 ^cells per well in RPMI-1640 supplemented with 25 mM HEPES, 26 mM NaHCO_3_, 2 mM L-glutamine, penicillin (100 units/ml), streptomycin (100 μg/ml), and 10% FBS and incubated overnight. Cells were then washed, incubated with 5 μM of the various FITC-labeled DPs, and electroporated with four-square pulses (7 V) of 60-milliseconds duration with 50-milliseconds intervals from a CUY21 pulse generator (Nepagene, Ichikawa, Chiba, Japan). After electroporation, cells were washed twice with medium and incubated at 37°C in 5% CO_2 _for 24 or 48 h.

### Western blotting

Cells in a 6-cm dish were washed twice with ice-cold phosphate buffered saline (PBS, 3.2 mM Na2HPO4, 0.5 mM KH2PO4, 1.3 mM KCl, 135 mM NaCl, pH 7.4) 24 h after transfection and then lysed in 200 μl of MLB buffer (25 mM HEPES, pH 7.5, 150 mM NaCl, 1% Igepal CA-630, 10 mM MgCl_2_, 1 mM EDTA, 10% glycerol) containing protease inhibitors (20 μg/ml aprotinin, 20 μg/ml phenylmethylsulfonyl fluoride) and phosphatase inhibitors (2 mM NaF, 1 mM Na_3_VO_4_). After centrifugation at 14,000 × *g *for 5 min, the proteins in the lysates were quantitated using the Bio-Rad Protein Assay Kit (Bio-Rad Laboratories, Inc., Hercules, CA). Proteins (50 μg) were separated on 15% SDS-polyacrylamide gels and transferred to polyvinylidene difluoride membranes. After the membranes had been blocked, they were incubated first with anti-myc (Invitrogen, Carlsbad, CA), anti-EGFP (Clontech), or anti-actin antibody (Sigma, St Louis, MO) for 12 h at 4°C and then with horseradish-peroxidase-conjugated goat anti-mouse IgG antibody at room temperature for 1 h. An ECL kit (Amersham, Buckinghamshire, UK) was used for the substrate reaction.

### Confocal microscopic analyses

Cells were plated on coverslips in six-well plates at 1 × 10^5 ^cells per well in RPMI-1640 containing 10% FBS and incubated overnight. The cells were then transfected with 1 μg of various RIG1 expression vectors for 18 h. The cells were washed and fixed with 4% paraformaldehyde and then analyzed with a Zeiss LSM510 laser scanning confocal microscope (Carl Zeiss Jena GmbH, Jena, Germany). Alternatively, the cells were electroporated with FITC-DPs for 48 h. The cells were washed, fixed, stained with 1 μg/ml 4'6-diamidino-2-phenylindole (DAPI), and then analyzed with a Leica TCS SP5 scanner (Leica, Bensheim, Germany). Analysis the expression of MRFP-tagged wild type and variants of RIG1 was the same as our previous study [12]. The BODIPY@FLC5-ceramide-BSA was used to stain trans-Golgi organelles. After stained with the DAPI dye, the cells were analyzed with a Leica TCS scanner.

### Analysis of cell death and apoptosis

Cells were plated in triplicate in 24-well plates at a density of 2 × 10^4 ^cells per well in RPMI-1640 medium containing 10% FBS and incubated overnight. The cells were transfected with 0.3 μg of various RIG1 expression vectors or empty control vector and then refreshed with complete medium immediately and 24 h after transfection. Cells were analyzed for viability using the MTT assay as described previously [12]. Cell viability relative to that of control transfected cells was defined as [(A570–A660) of RIG1-transfected cells/(A570–A660) of control transfected cells] × 100%. Measurement of the release of LDH using the Cytotoxicity Detection Kit (Roche Molecular Biochemicals, Mannheim, Germany) was used to evaluate cell death as described previously [12]. Percentage LDH release was defined as [(A490–A650) of RIG1-cells/(A490–A650) of control transfected cells] × 100%. Chromatin condensation was used as a measure of cellular apoptosis. Briefly, cells in 6-cm dishes were transfected with various plasmids for 48 h and then fixed with 4% paraformaldehyde. After the cells were washed twice with cold PBS, they were incubated at room temperature for 10 min with PBS containing 1 μg/ml Hoechst 33258 and 4% bovine serum albumin. Chromatin condensation was analyzed with an Olympus IX-70 immunofluorescent microscope (Olympus, Glostrup, Denmark).

### Measurement of caspase 3 activity

HtTA cells were transfected with recombinant RIG1 expression vectors or electroporated with wild type or mutated DPs for 24 h. Cells were lysed in MLB containing protease and phosphatase inhibitors. To measure the activities of caspase-3, cell lysates were incubated at room temperature for 12 h with buffer containing 25 mM HEPES, pH 7.5, 0.1% CHAPS, 5% sucrose, 5 mM DTT, 2 mM EDTA, and 2 mM substrate for caspase-3 (Ac-DEVD-pNA; Sigma). Absorbance at 405 nm was measured with a microplate reader.

## Abbreviations

DAPI: 4'6-diamidino-2-phenylindole; DP: dodecapeptide; EGFP: enhanced green fluorescence protein; FBS: fetal bovine serum; FITC: fluorescein; MRFP: monomer red fluorescence protein; LRAT: lecithin:retinol acyltransferase; PBS: phosphate buffered saline; RIG1: Retinoid-inducible gene 1; RIG1_111–123_: corresponding to amino acids 112–123 located within the NC domain of RIG1.

## Authors' contributions

FMT performed most studies, contributed to experimental designed and drafted the manuscript. RYS contributed to experimental designed. SCL contributed to vector construction and performed some experiments. CCW contributed to experimental designed. SYJ designed and supervised the experiments and revised the manuscript. All authors were involved in the conception of the study and data interpretation. All authors read and approved the final version of the manuscript.

## Supplementary Material

Additional file 1 **Subcellular localization of wild-type and truncated MRFP-RIG1 fusion proteins.** HtTA cells were transiently transfected with indicated MRFP-RIG1 expression vector for 18 h. Cells were incubated with trans-Golgi-specific dye, stained with DAPI and then analyzed with a laser scanning confocal microscope. Scale bar: 5 μm. Click here for file

Additional file 2 **Analysis of the expression of EGFP-tagged DP variants of RIG1**. **(A) **Western blot analysis of the EGFP-DPs. HtTA cells plated in 6-cm dishes were transiently transfected for 24 h with 1.5 μg of indicated expression vector for EGFP or EGFP-DPs. (**B) **Subcellular localization of EGFP-DP fusion proteins. After HtTA cells were transiently transfected with EGFP-DP expression vector for 18 h, cells were fixed and analyzed with a laser scanning confocal microscope. Scale bar: 5 μm. Click here for file

Additional file 3 **Analysis of the effect of EGFP-DP fusion proteins on cell death of HtTA cells**. **(A) **EGFP-DP fusion proteins induced cell death. HtTA cells were transfected with 0.3 μg of the indicated expression vectors for the EGFP-DP fusion protein for 24 or 48 h. Cell death was detected by measuring LDH release, and cell viability was measured with the MTT method. Representative results of three independent experiments are shown and are expressed as means and standard errors of the means after normalization to the control EGFP group. Student *t *test: *, P < 0.05; **, P < 0.01. Cells were transfected with expression vectors for the EGFP **(B)**, or various mutated DP fusion proteins **(C) **for 48 h. Cells were stained with Hoechst 33258 dye, and chromatin condensation was evaluated with a fluorescent microscope. Arrows indicate cells expressing the EGFP-DP fusion protein that were positive for chromatin condensation. Arrowheads indicate mitotic cells. Scale bar: 1 μm. Click here for file

Additional file 4 **Expression vectors**. Detailed procedures for the construction of expression plasmids that synthesized recombinant proteins containing wild type or mutated DPs tagged to the C-terminus of EGFP or MRFP. Click here for file
