# Supplementary figures and images for: Induction of apoptosis by the retinoid inducible growth regulator RIG1 depends on the NC motif in HtTA cervical cancer cells
Source: BMC Cell Biol. 2009 Feb 26;10:15. doi: 10.1186/1471-2121-10-15 (PMC2656461; doi:10.1186/1471-2121-10-15)

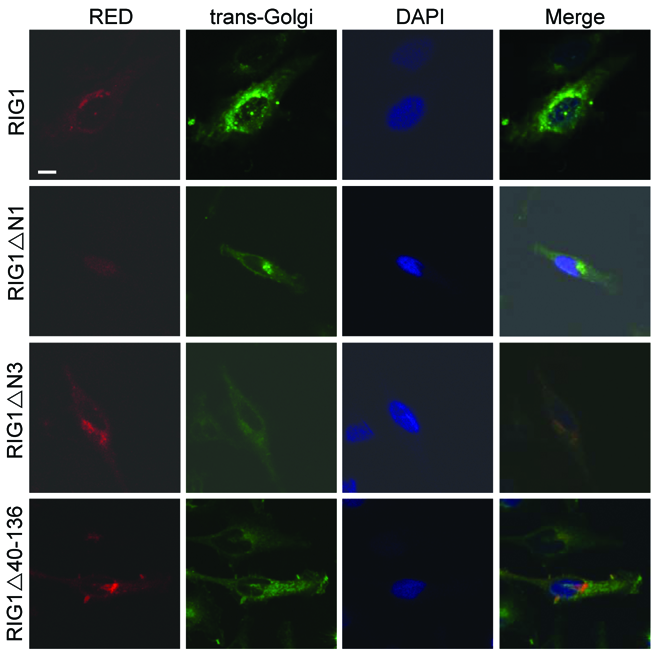

Supplement: Additional file 1 — Subcellular localization of wild-type and truncated MRFP-RIG1 fusion proteins. HtTA cells were transiently transfected with indicated MRFP-RIG1 expression vector for 18 h. Cells were incubated with trans-Golgi-specific dye, stained with DAPI and then analyzed with a laser scanning confocal microscope. Scale bar: 5 μm. [file 1471-2121-10-15-S1.tiff]

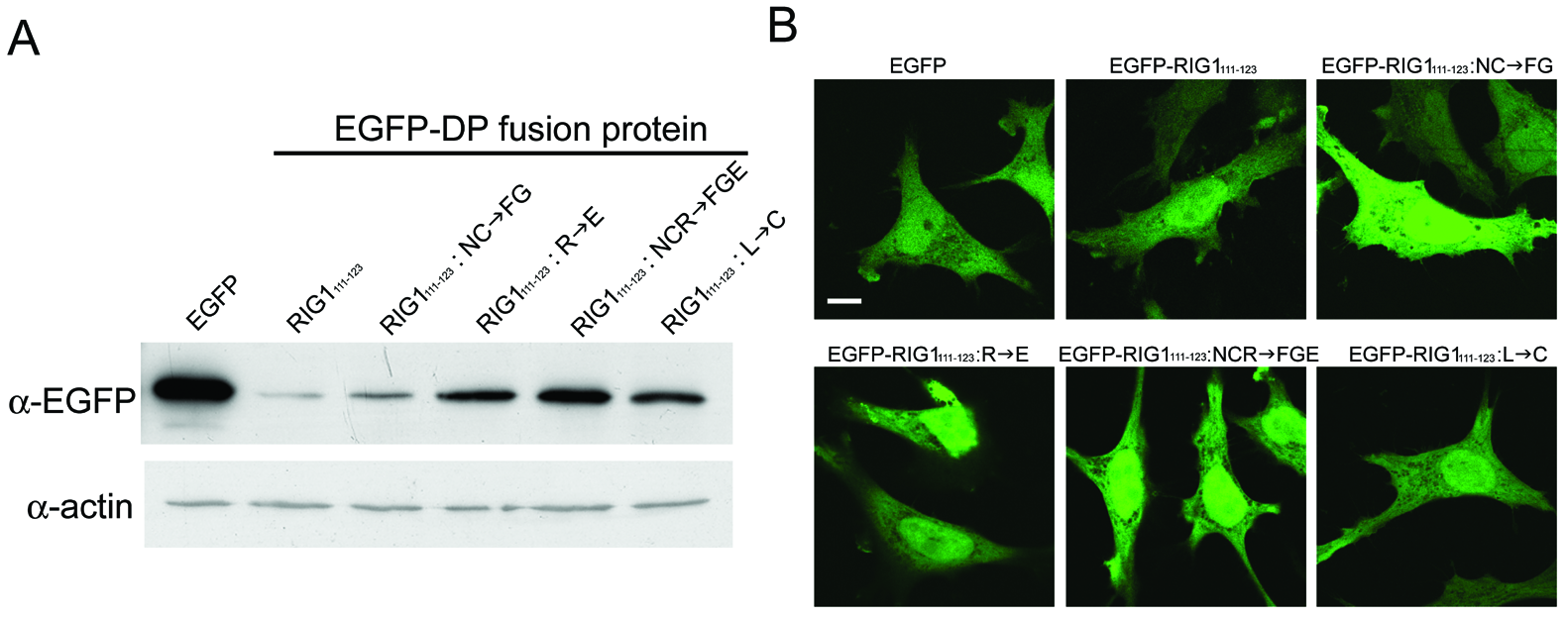

Supplement: Additional file 2 — Analysis of the expression of EGFP-tagged DP variants of RIG1. (A) Western blot analysis of the EGFP-DPs. HtTA cells plated in 6-cm dishes were transiently transfected for 24 h with 1.5 μg of indicated expression vector for EGFP or EGFP-DPs. (B) Subcellular localization of EGFP-DP fusion proteins. After HtTA cells were transiently transfected with EGFP-DP expression vector for 18 h, cells were fixed and analyzed with a laser scanning confocal microscope. Scale bar: 5 μm. [file 1471-2121-10-15-S2.tiff]

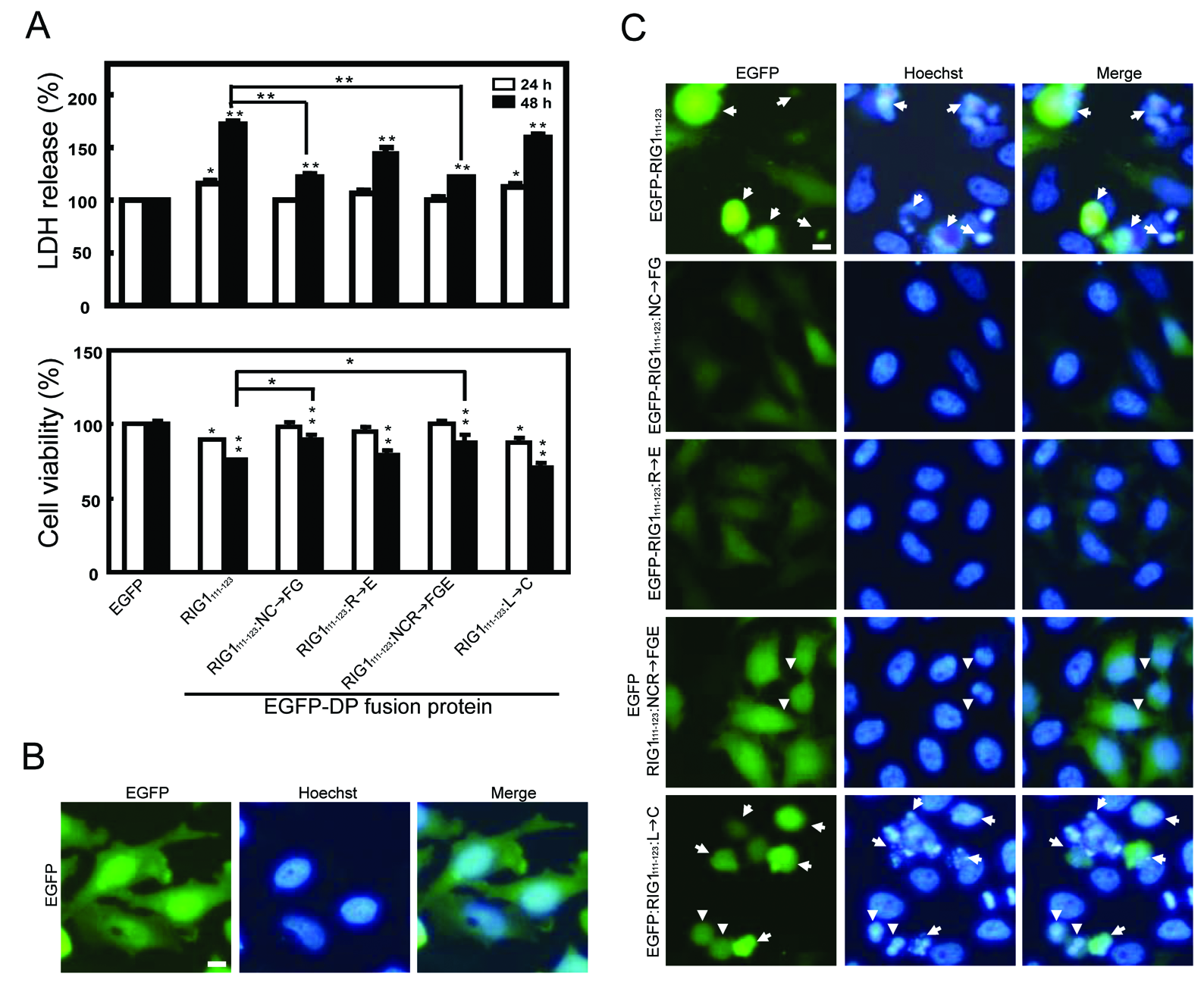

Supplement: Additional file 3 — Analysis of the effect of EGFP-DP fusion proteins on cell death of HtTA cells. (A) EGFP-DP fusion proteins induced cell death. HtTA cells were transfected with 0.3 μg of the indicated expression vectors for the EGFP-DP fusion protein for 24 or 48 h. Cell death was detected by measuring LDH release, and cell viability was measured with the MTT method. Representative results of three independent experiments are shown and are expressed as means and standard errors of the means after normalization to the control EGFP group. Student t test: *, P < 0.05; **, P < 0.01. Cells were transfected with expression vectors for the EGFP (B), or various mutated DP fusion proteins (C) for 48 h. Cells were stained with Hoechst 33258 dye, and chromatin condensation was evaluated with a fluorescent microscope. Arrows indicate cells expressing the EGFP-DP fusion protein that were positive for chromatin condensation. Arrowheads indicate mitotic cells. Scale bar: 1 μm. [file 1471-2121-10-15-S3.tiff]
