# Supplementary material for: Induction of apoptosis by the retinoid inducible growth regulator RIG1 depends on the NC motif in HtTA cervical cancer cells
Source: BMC Cell Biol. 2009 Feb 26;10:15. doi: 10.1186/1471-2121-10-15 (PMC2656461; doi:10.1186/1471-2121-10-15)
Supplement: Additional file 4 — Expression vectors. Detailed procedures for the construction of expression plasmids that synthesized recombinant proteins containing wild type or mutated DPs tagged to the C-terminus of EGFP or MRFP. [file 1471-2121-10-15-S4.pdf]

Additional File 4

Expression vectors. To generate expression plasmids that synthesized recombinant proteins containing wild type or mutated DPs tagged to the C-terminus of EGFP, primer pairs specific for $RIG1_{111-123}$ (5'-TCGAGTTAGGAACTGTGAGCACTTTGTCACCCAGCTGAGATATGGCTGAG-3' and 5'-AATTCTCAGCCATATCTCAGCTGGGTGACAAAGTGACAGTTCCTAAC-3'), the NC-mutated DP ($RIG1_{111-123}$:NC→FG) (5'-TCGAGTTAGGTTTGGCGAGCACTTTGTCACCCAGCTGAGATATGGCTGAG-3' and 5'-AATTCTCAGCCATATCTCAGCTGGGTGACAAAGTGCTCGCCAAACCTAAC-3'), the Arg^{121}-mutated DP ($RIG1_{111-123}$:R→E) (5'-TCGAGTTAGGAACTGTGAGCACTTTGTCACCCAGCTGGAGTATGGCTGAG-3' and 5'-AATTCTCAGCCATACTCCAGCTGGGTGACAAAGTGCTCACAGTTCCTAAC-3'), the $Asp^{112}Cys^{113}Arg^{121}$-mutated DP ($RIG1_{111-123}$:NCR→FGE) (5'-TCGAGTTAGGTTTGGCGAGCACTTTGTCACCCAGCTGGAGTATGGCTGAG-

3’ and

5’-AATTCTCAGCCATACTCCAGCTGGGTGACAAAGTGCTCGCCAAACCTAAC-3

’) and the Leu^{120}-mutated DP ($RIG1_{111\text{-}123}$:L→C)

(5’-TCGAGTTAGGAACTGTGAGCACTTTGTCACCCAGTGTAGATATGGCTGAG-3

’ and

5’-AATTCTCAGCCATATCTACACTGGGTGACAAAGTGCTCACAGTTCCTAAC-3’)

were annealed, and then subcloned in-frame into *Xho*I-*Eco*RI sites of the pEGFP-C1. Seven amino acids (Ser-Gly-Leu-Arg-Ser-Arg-Val) were inserted between EGFP and DP to serve as a linker, and synthesis of EGFP-DP fusion variants was terminated immediately after the sequence of DP. Expression vectors that synthesized MRFP-tagged truncated RIG1 variants were constructed by subcloned respective cDNA fragment into the pDsRED-Monomer-C1 (Cloned Laboratories).
